# Cortical Activation During Finger Tapping Task Performance in Parkinson's Disease Is Influenced by Priming Conditions: An ALE Meta-Analysis

**DOI:** 10.3389/fnhum.2021.774656

**Published:** 2021-11-30

**Authors:** Jingjing Li, Zheng Liu, Zhongquan Du, Ningning Zhu, Xueqing Qiu, Xia Xu

**Affiliations:** ^1^Graduate School, Wuhan Sports University, Wuhan, China; ^2^ANU College of Health and Medicine, Australian National University, Canberra, ACT, Australia; ^3^Sydney School of Education and Social Work, University of Sydney, Sydney, NSW, Australia; ^4^College of Health Science, Wuhan Sports University, Wuhan, China; ^5^Hubei Key Laboratory of Exercise Training and Monitoring, Wuhan Sports University, Wuhan, China

**Keywords:** finger tapping task, Parkinson's disease, motor control, self-priming, cue-priming, ALE meta-analysis

## Abstract

The finger tapping task (FTT) is commonly used in the evaluation of dyskinesia among patients with Parkinson's disease (PD). Past research has indicated that cortical activation during FTT is different between self-priming and cue-priming conditions. To evaluate how priming conditions affect the distribution of brain activation and the reorganization of brain function, and to investigate the differences in brain activation areas during FTT between PD patients and healthy control (HC) participants, we conducted an activation likelihood estimation (ALE) meta-analysis on the existing literature. Analyses were based on data from 15 independent samples that included 181 participants with PD and 164 HC participants. We found that there was significantly more activation in the middle frontal gyrus, precentral gyrus, post-central gyrus, superior parietal lobe, inferior parietal lobule, cerebellum, and basal ganglia during FTT in PD patients than in HCs. In self-priming conditions, PD patients had less activation in the parietal lobe and insular cortex but more activation in the cerebellum than the HCs. In cue-priming conditions, the PD patients showed less activation in the cerebellum and frontal-parietal areas and more activation in the superior frontal gyrus and superior temporal gyrus than the HCs. Our study illustrates that cue-priming manipulations affect the distribution of activity in brain regions involved in motor control and motor performance in PD patients. In cue-priming conditions, brain activity in regions associated with perceptual processing and inhibitory control was enhanced, while sensory motor areas associated with attention and motor control were impaired.

## Introduction

Parkinson's disease (PD) is a common neurodegenerative disease among middle-aged and elderly people. Its clinical features mainly include bradykinesia, rigid muscles, static tremor, postural instability, and gait disorders (Marsden, 1994; Tolosa et al., 2009; for reviews on the history of PD, see Goetz, 2011). These symptoms have significant influences on one's survival, quality of life, and nursing home placement demand. The incidence rate of PD in elderly individuals aged over 60–65 years is over 1–2%, and the prevalence increases with age (Hirtz et al., 2007; Elbaz et al., 2016).

Bradykinesia is one of the most common and serious symptoms of PD and is most obviously shown during movement repetition (Bologna et al., 2016). PD patients usually have reduced movement, speed, and amplitude and have difficulties in autonomous movement (Sethi, 2008). The finger tapping task (FTT, also known as a tapping speed assessment) has been one of the most commonly paradigms that used to assess muscle control and motor ability since the nineteenth century (Golden et al., 2002; Picillo et al., 2016). The original FTT paradigm required participants to place their palms flat on a surface and continuously tap with their index finger in blocks of 10–30 s. The number of strokes by a participant was recorded. Over the years, the paradigm has been modified into different forms to suit various conditions (Levit-Binnun et al., 2007; Versaci and Laje, 2021). FTT is affected by many factors including hand dominance, age, gender, and neural control. Neuroimaging studies suggest that the primary motor area of the hand and the cerebellum plays a pivotal role in the control of finger tapping (Jancke et al., 2004). The performance of finger tapping is related to neural mechanisms located in supra-spinal structures (Studenka and Zelaznik, 2011), Normal finger tapping requires the functional integrity of the corticospinal tract, cerebellar motor circuitry, and proprioceptive pathways (Zhang et al., 2018). The usefulness of the finger tapping task for specific PD motor assessment has been proven by evidences showing correlation with the extent of loss of neurons in the substantial nigra, assessed *in vivo* with [18F]-6-fluoro-L-dopa (6-FD) PET (Pal et al., 2001). The FTT is an effective evaluation index of movement impairments such as bradykinesia in PD patients. When performing the FTT, PD patients tend to show lower tapping speed and a decreased range of motion (Lee et al., 2010; Stegemöller et al., 2015). Owing to their abnormal basal ganglia output, PD patients lack the finer cortical control and greater facilitation that the finger task demands.

For the development of treatment methods, a better understanding of the neurological basis of PD symptoms is crucial. Previous structural and functional imaging investigations have made preliminary attempts to elucidate the neural basis of bradykinesia in PD patients (Baudrexel et al., 2011; Ziegler et al., 2014; Hirano, 2021). As the FTT has the advantage of being simple and flexible enough to use in the study of both PD patients and healthy controls (HCs), it is often used in functional neuroimaging studies to evaluate the integrity of motor control and neuromuscular system function in PD patients (Witt et al., 2008; Wurster et al., 2015). In recent years, a large number of neuroimaging studies have discovered changes in brain activation during FTT performance in PD patients (Wurster et al., 2015; Bologna et al., 2016). Studies have reported that the structures activated in PD patients during the FTT include the primary motor cortex, supplementary motor area (Jia et al., 2018), parietal lobe (Samuel et al., 1997; Tessa et al., 2013), ventrolateral thalamic nucleus (Mallol et al., 2007), and inferior frontal gyrus (Disbrow et al., 2013). Some studies have refined the activated brain areas to the superior temporal gyrus, inferior frontal gyrus (Cerasa et al., 2006) and middle frontal gyrus (Mak et al., 2016). Areas reported in previous studies to show possible impairments include the sensorimotor cortex (Georgiou et al., 1994; Martin et al., 2019), basal ganglia (Witt et al., 2008; Liberg et al., 2013; Ruppert et al., 2020), thalamus (Samuel et al., 1997; Mak et al., 2016; Jia et al., 2018) and cerebellum (Rowe et al., 2002; Cerasa et al., 2006; del Olmo et al., 2006). Other areas that exhibited insufficient activation are the putamen (Martin et al., 2019), superior parietal lobule, insula cortex (Wu and Hallett, 2005), and striatum (Wu et al., 2011). Some studies have also found insufficient activation in the supplementary motor area (Martin et al., 2019) and primary motor cortex (Hughes et al., 2010). In contrast to impairments, some studies reported greater activation of the primary motor cortex (Yan et al., 2015) and cerebellar regions (Rowe et al., 2002; Yan et al., 2015; Mirdamadi, 2016) during FTT performance in PD patients than in HCs, suggesting possible compensatory connectivity mechanisms in PD.

In summary, the distribution of activated areas and the direction of activation changes in existing studies have been inconsistent (Levit-Binnun et al., 2007; Witt et al., 2008), thus providing an ambiguous picture of the pathophysiological mechanisms of motor control in PD patients. It is difficult to identify clear brain activation patterns of PD patients during FTT performance with these inconsistent and even contradictory neural findings. One reason for the inconsistencies is the use of different priming conditions during the FTT (Georgiou et al., 1994; Lim et al., 2005). Past research has shown that PD patients rely more strongly on external cues in motor control than HC participants (Georgiou et al., 1994; Mak and Hui-Chan, 2004, 2008). The external cues can be audio cues (e.g., regular rhythms generated by a metronome) (Hackney et al., 2015; Qureshi et al., 2020), visual cues (e.g., flashing lights) (van Eimeren et al., 2006), etc. The FTT condition that is performed in the presence of an external cue is called cue-priming or cue-initiated tapping. The FTT condition that is performed without an external cue is called self-priming or self-initiated tapping. Past studies have indicated that priming manipulations may affect the motor control and motor output of individuals with PD (Morris et al., 1994; Mak et al., 2016), thus impacting the distribution of activity across brain regions.

The small sample sizes and the heterogeneity in sample characteristics in neuroimaging studies also contribute to inconsistencies (Gottlieb and Oudeyer, 2018; Raut et al., 2019). Thus, there is a need for a more systematic approach to integrate the existing results and to accurately describe the brain activation patterns during FTT performance (Poldrack et al., 2011). Although some recent reviews have investigated some of the functional mechanisms underlying motor symptoms in PD (Mirdamadi, 2016; Tahmasian et al., 2017), no quantitative review has focused on the impact of different priming conditions on the distribution of brain region activation in PD. Powerful meta-analysis can be aggregate prior studies together to analyze the characteristics of brain activation in PD patients performing the FTT under different priming conditions, which also makes it possible to compare the brain activation differences between PD and HC participants under the same priming conditions. Therefore, the present study used activation likelihood estimation (ALE) meta-analysis, which is a quantitative meta-analytic method that has been widely utilized to determine the stereoscopic brain coordinates that have been consistently active across studies (Turkeltaub et al., 2002; Acar et al., 2018). With an ALE analysis, we aimed to study the influence of priming conditions on brain activation during FTT performance in PD patients and to analyze the influence of cue priming on brain activity and functional reorganization ofPD patients. In this way, we hope to contribute to the current understanding of the functional differentiation of brain areas involved during FTT performance in PD patients, which has practical implications for designing future interventions for their treatment.

## Materials and Methods

### Literature Search and Inclusion Criteria

The current meta-analysis utilized anonymity data so that it was exempt from approval by the ethics committee of the authors' institution.

After determining the research topic, a systematic search was conducted following the Preferred Reporting Items for Systematic Reviews and Meta-Analyses (PRISMA) guidelines (Moher et al., 2009). The targeted online databases were PubMed, ISI Web of Science, and EBSCO Academic Resource Retrieval Center (ASP and BSP). The search terms were (“Parkinson's disease” OR “Parkinson's disease” OR “Parkinson's disease”) AND (“functional magnetic resonance” OR “fMRI” OR “positron emission tomography” OR “PET”) AND (“tap” OR “tapping” OR “finger” OR “finger tap” OR “finger tapping” OR “Motor control”). To identify papers that might have been missed, several other sources were screened, including the citation index of the BrainMap database (http://BrainMap.org), pre-print articles (https://psyarxiv.com/) and their reference lists. The first search was carried out on July 1st, 2020. The broad search yields 1,358 articles.

**Figure 1:**
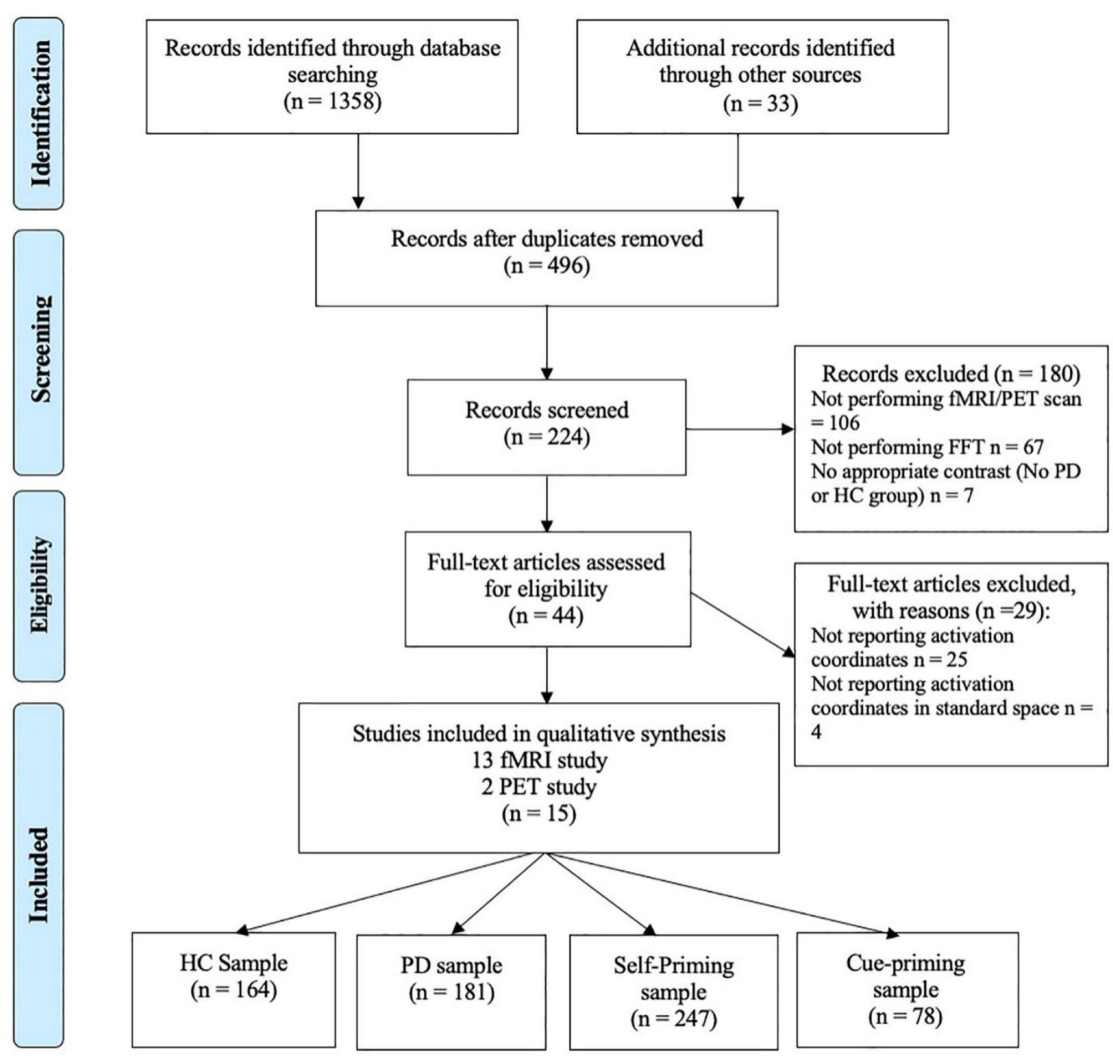
PRISMA flow diagram depicting identification, screening, and inclusion strategies for the selection of the reviewed studies.

After deleting duplicate studies, 496 articles remained. All articles were assessed by two independent raters to decide on the eligibility of studies. Weekly consensus meetings were held, and only articles that both raters agreed on were included in the meta-analysis. A total of 224 articles were determined to be relevant to the topic of this study. By examining the abstracts of the articles, 44 articles were able to proceed to full-text review according to the inclusion criteria and exclusion criteria. The inclusion criteria were as follows: (1) the original research paper was published in a peer-reviewed journal; (2) participants included at least one PD group and one HD group contrast; (3) descriptive statistics of the FTT were reported; and (4) fMRI or PET scans were performed. The exclusion criteria were as follows: (1) severity of the disease (UPDRS score^1^or H&Y score^2^) was not reported; (2) no specific activation coordinates were reported; and (3) activation coordinates were not in standard space (MNI or Talairach). After applying the exclusion and inclusion criteria, a final set of 15 papers (13 fMRI studies, 2 PET studies), including a total of 181 PD patients and 164 HC participants, were included in the final analysis. The detailed retrieval process is shown in Figure 1.

### Coding of Studies

Two independent coders extracted the basic information (authors, year of publication) for all included studies and compiled a table of study characteristics based on FTT pacing conditions (self-priming or cue-priming), imaging methods, sample size, mean age, and UPDRS scores. A third coder was responsible for the verification. Inconsistencies were rereviewed by the three coders together until consensus was reached. After coding all the targeted elements, a full listing of the studies included in the meta-analyses and their corresponding demographic information was compiled and is shown in Table 1.

**Table 1:** Characteristics of the studies included in the meta-analysis.

**No**.	**Experiment**	**Pacing type**	**Imaging method**	**Number of PD participants**	**Number of HC participants**	**UPDRS off score**	**UPDRS on score**	**Mean age PD**	**Mean age HC**
1	Samuel et al., 1997	Self-priming^1^	PET	6	6	17.7		70.2	64.3
	Samuel et al., 1997	Self-priming^2^	PET	6	6	17.7		70.2	64.3
2	Sabatini et al., 2000	Self-priming	fMRI	6	6	16		61	59
3	Rowe et al., 2002	Cue-priming	fMRI	12	12	33.7		62	62
4	Wu and Hallett, 2005	Self-priming	fMRI	12	12	25.5		61.2	61.8
5	Cerasa et al., 2006	Cue-priming	fMRI	10	11	27.5		62.4	63.4
	Cerasa et al., 2006	Self-priming	fMRI	10	11	27.5		64.2	63.4
6	Mallol et al., 2007	Self-priming	fMRI	13	11	22.6		64.9	61.9
7	Hughes et al., 2010	Self-priming	fMRI	16	15	31.3	18.9	63.9	66.5
8	Wu et al., 2011	Self-priming^1^	fMRI	15	15	20.7		59.7	60.3
	Wu et al., 2011	Self-priming^2^	fMRI	15	15	20.7		59.7	60.3
9	González-García et al., 2011	Cue-priming	fMRI	17	10		41	64.4	
	González-García et al., 2011	Self-priming	fMRI	17	10		41	64.4	
10	Disbrow et al., 2013	Cue-priming	fMRI	13	13			57.5	63.9
11	Tessa et al., 2013	Self-priming	fMRI	11	10	13.5		67.7	64.0
12	Yan et al., 2015	Cue-priming	fMRI	11	12	20.1		61.5	65.5
13	Mak et al., 2016	Self-priming	fMRI	27	28	29.0		61.4	60.9
	Mak et al., 2016	Cue-priming	fMRI	27	28	29.0		61.4	60.9
14	Jia et al., 2018	Self-priming	fMRI	22	22	16.5		61.0	60.6
15	Martin et al., 2019	Self-priming	fMRI	22	22	19.6		53.0	48.5

*Superscripts ^1, 2^ marked in the pacing-type section refer to different forms of the finger tapping tasks used within the same article; fMRI, functional magnetic resonance imaging; PET, positron emission tomography; UPDRS, Unified Parkinson's Disease Rating Scale; the off/on score denotes the scale score in off/on periods. On phase, the period of maximum efficacy of dopaminergic drugs; OFF phase, the period when PD symptoms appear again*.

### Meta-Analytic Procedure

For the analysis, the ALE method is used. It is one of the most commonly used methods for meta-analysis across different brain imaging studies (Turkeltaub et al., 2002). The ALE analysis assumes that for every study of interest, there must be a given spatial distribution of activity and a set of associated maximum coordinates (Laird et al., 2009). In this study, we followed the algorithm proposed by Turkeltaub et al. (2012). It provides a means of evaluating this hypothesis within the framework of a permutation test and is thus able to pinpoint areas of the brain that are more reliably activated across studies. All ALE analyses were run using GingerALE 3.0.2 (http://www.brainmap.org/ale/). The coordinates of brain activation regions were extracted from the original papers. To allow for direct comparisons of spatial brain coordinates across studies, relevant foci were converted from the Talairach coordinates into MNI coordinates using the Lancaster transform (Lancaster et al., 2007). These foci are modeled as the center of a three-dimensional Gaussian probability distribution. With a cluster threshold of *k* = 120 mm^3^, all individual analyses were adjusted for multiple comparisons using the false discovery rate (FDR) *p* < 0.01 with 5,000 permutations (Turkeltaub et al., 2002, 2012; Laird et al., 2010). The resulting ALE maps were overlaid on the Colin brain template in MNI space and visualized by Mango 4.1 (rii.uthscsa.edu/mango) (Eickhoff et al., 2012). The coordinate points beyond the template boundary were uniformly deleted.

## Results

### Description of the Demographic Information

Fifteen studies (13 fMRI, 2 PET) were included in the ALE meta-analysis. Twenty experiments were reported, with a total sample size of 345 people (181 PD patients). The experiments were divided into four subgroups: the PD self-priming group, PD cue-priming group, HC self-priming group, and HC cue-priming group. Study characteristics and participant demographics are summarized in Table 1. The UPDRS (ON) score of the PD participants included in the study was 22.86 ± 5.97, which was equivalent to stages I-II of the Hoehn-Yahr scale, indicating mild to moderate disease severity of the participants. There was no significant age difference between the PD group (*M*_*age*_= 62.29, *SD*_*age*_ = 4.00) and HC group (*M*_*age*_= 61.75, *SD*_*age*_= 3.88) (*t* = 0.83, *p* = 0.42).

### ALE Meta-Analysis Results

An ALE analysis was used to determine the cluster of the most significant activation points in the brain activation map and activation regions during finger tapping. The anatomical location and Brodmann area (BA) of each cluster was defined. In the 20 experiments included in the analysis, a total of 160 activation points were identified during performance of the FTT. The largest clusters of activation were located in the middle frontal gyrus, precentral rgyrus, post-central gyrus, superior parietal lobe, inferior parietal lobule, cerebellum, and basal ganglia. These regions are considered the core regions involved in FTT performance in the PD patients.

#### ALE Meta-Analysis Results With the PD Group in Different Priming Conditions

**Figure 2:**
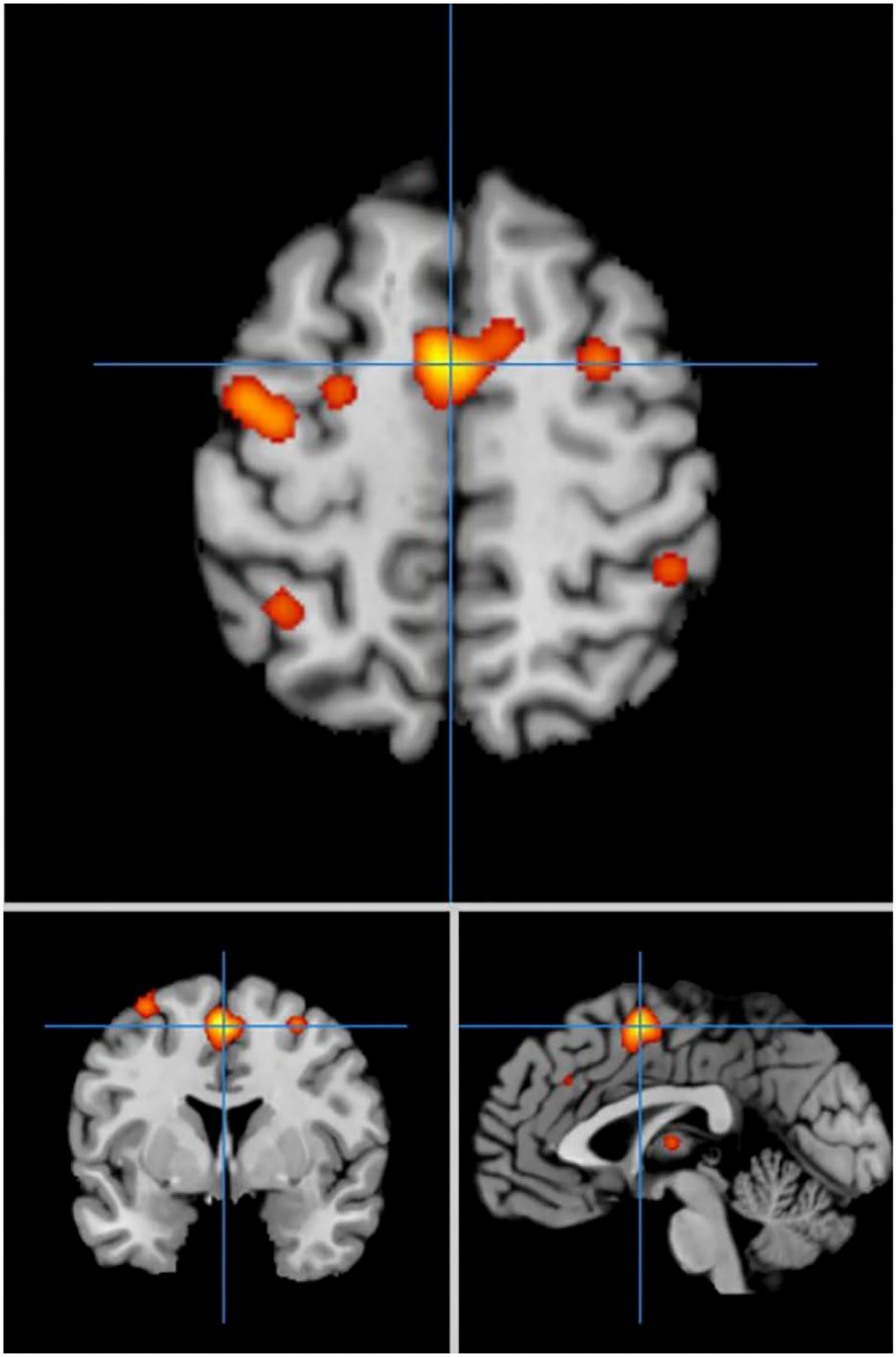
Significant clusters in the ALE meta-analysis in self-priming conditions in the PD patients.

In self-priming conditions, a total of 74 PD patients in 7 experiments were included in the meta-analysis, obtaining 61 activation points (Figure 2). There were 17 clusters of activation, which were located in the cerebellum; both sides of the middle frontal gyrus, cingulate gyrus, medial frontal gyrus, and post-central gyrus; the right side of the superior frontal gyrus, transverse temporal gyrus, inferior parietal lobule, and precuneus; and the left side of the superior temporal gyrus, thalamus, and superior parietal lobule (Table 2).

**Table 2:** Clusters of activation in self-priming conditions in the PD patients.

**Activation cluster**	**Anatomical region**		**BA Area**	**x**	**y**	**z**	**ALE**
#1	R	Superior frontal gyrus	BA 8	38	32	50	0.005
#2	R	Middle frontal gyrus	BA 6	38	−6	46	0.005
#3	L	Middle frontal gyrus	BA 6	−24	−4	56	0.0072
#4	R	Transverse temporal gyrus	BA 41	58	−22	10	0.0065
#5	R	Cingulate gyrus	BA 32	10	30	42	0.005
#6	L	Cingulate gyrus	BA 32	−2	30	32	0.0049
#7	R	Medial frontal gyrus	BA 6	10	6	56	0.0069
#8	L	Medial frontal gyrus	BA 6	−2	0	56	0.0109
#9	L	Superior temporal gyrus	BA 41	−60	−24	14	0.0068
#10	R	Middle temporal gyrus	BA 21	66	−46	−2	0.0079
#11	L	Thalamus		−4	−12	6	0.0069
#12	L	Superior parietal lobule	BA 40	−36	−50	56	0.0069
#13	R	Inferior parietal lobule	BA 40	40	−44	44	0.0051
#14	R	Cerebellum		40	−64	−28	0.007
#15	R	Precuneus	BA 7	24	−66	46	0.0069
#16	R	Post-central gyrus	BA 2	54	−28	40	0.0074
#17	L	Post-central gyrus	BA 2	−42	−20	48	0.0071

*R, right hemisphere; L, left hemisphere; BA, Brodmann Area*.

**Figure 3:**
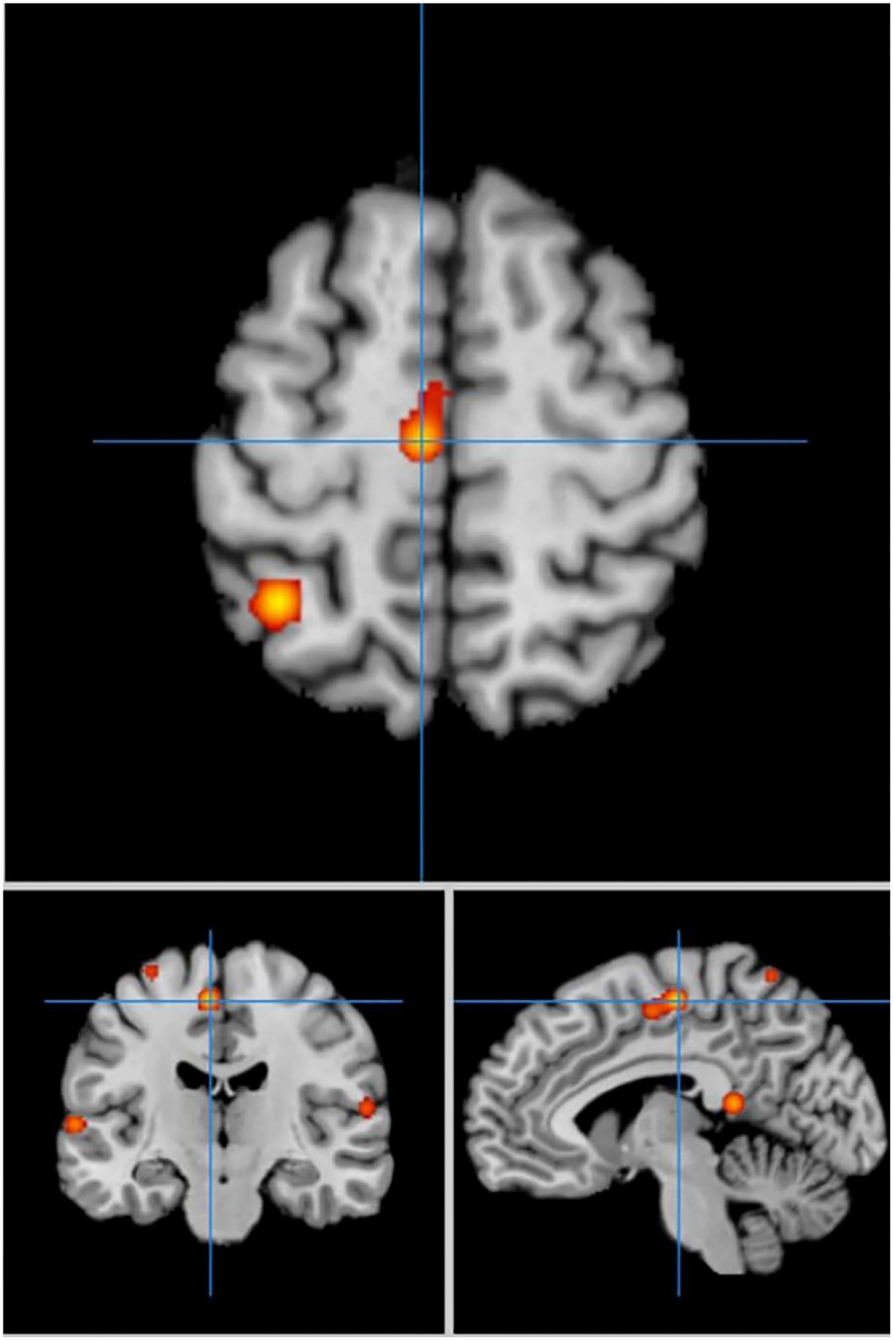
Significant clusters in the ALE meta-analysis in cue-priming conditions in the PD patients.

In cue-priming conditions, a total of 33 PD participants in 3 experiments were included in the meta-analysis, obtaining 28 activation points (Figure 3). There were 21 clusters of activation, which were located on both sides of the inferior frontal gyrus, middle frontal gyrus, middle temporal gyrus, superior parietal lobe, posterior lobe of cerebellum VI, cerebellum, and pre-central gyrus; the left side of the precuneus, medial frontal gyrus, superior temporal gyrus, posterior cingulate cortex, and ventrolateral thalamic nucleus; and the right side of the transverse temporal gyrus and lingual gyrus (Table 3).

**Table 3:** Clusters of activation in cue-priming conditions in the PD patients.

**Activation cluster**	**Anatomical region**		**BA area**	**x**	**y**	**z**	**ALE**
#1	L	Precuneus	BA 7	−10	−58	66	0.0069014
#2	R	Inferior frontal gyrus	BA 47	52	32	−12	0.0065224
#3	L	Inferior frontal gyrus	BA 45	−54	26	0	0.0065673
#4	R	Middle frontal gyrus	BA 6	46	6	38	0.0069025
#5	L	Middle frontal gyrus	BA 9	−30	38	18	0.0069257
#6	R	Transverse temporal gyrus	BA 41	60	−22	10	0.0064963
#7	L	Medial frontal gyrus	BA 6	−6	−18	56	0.0070025
#8	L	Superior temporal gyrus	BA 41	−62	−20	4	0.0063104
#9	R	Middle temporal gyrus	BA 37	60	−60	4	0.0069014
#10	L	Middle temporal gyrus	BA 37	−52	−58	8	0.0069014
#11	L	Ventrolateral thalamic nucleus		−12	−12	4	0.0065658
#12	R	Superior parietal lobule	BA 7	44	−52	62	0.0069033
#13	L	Superior parietal lobule	BA 7	−38	−54	62	0.0079067
#14	L	Posterior cingulate cortex	BA 29	−4	−42	12	0.0069014
#15	R	Posterior lobe of cerebellum VI		48	−62	−22	0.0070793
#16	L	Posterior lobe of cerebellum VI		−50	−68	−18	0.0067592
#17	L	Anterior lobe		−18	−52	−30	0.0069014
#18	R	Anterior lobe		22	−54	−28	0.0069014
#19	R	Lingual gyrus	BA 18	14	−92	−8	0.0069014
#20	R	Pre-central gyrus	BA 4	60	−2	44	0.0064317
#21	L	Pre-central gyrus	BA 4	−30	−22	68	0.0065661

Comparing the activated regions between the self-priming and cue-priming conditions, the middle frontal gyrus, pre-central gyrus, superior parietal lobule, precuneus areas, cerebellum and basal ganglia were consistently activated to a greater extent. In self-priming conditions, the superior frontal gyrus, post-central gyrus, and cingulate gyrus were activated. Comparatively, in cue-priming conditions, the temporal cortex, anterior lobe, precentral gyrus, inferior frontal gyrus and lingual gyrus appeared to be activated.

#### Comparisons Between the PD and HC Groups in Self-Priming Conditions

**Figure 4:**
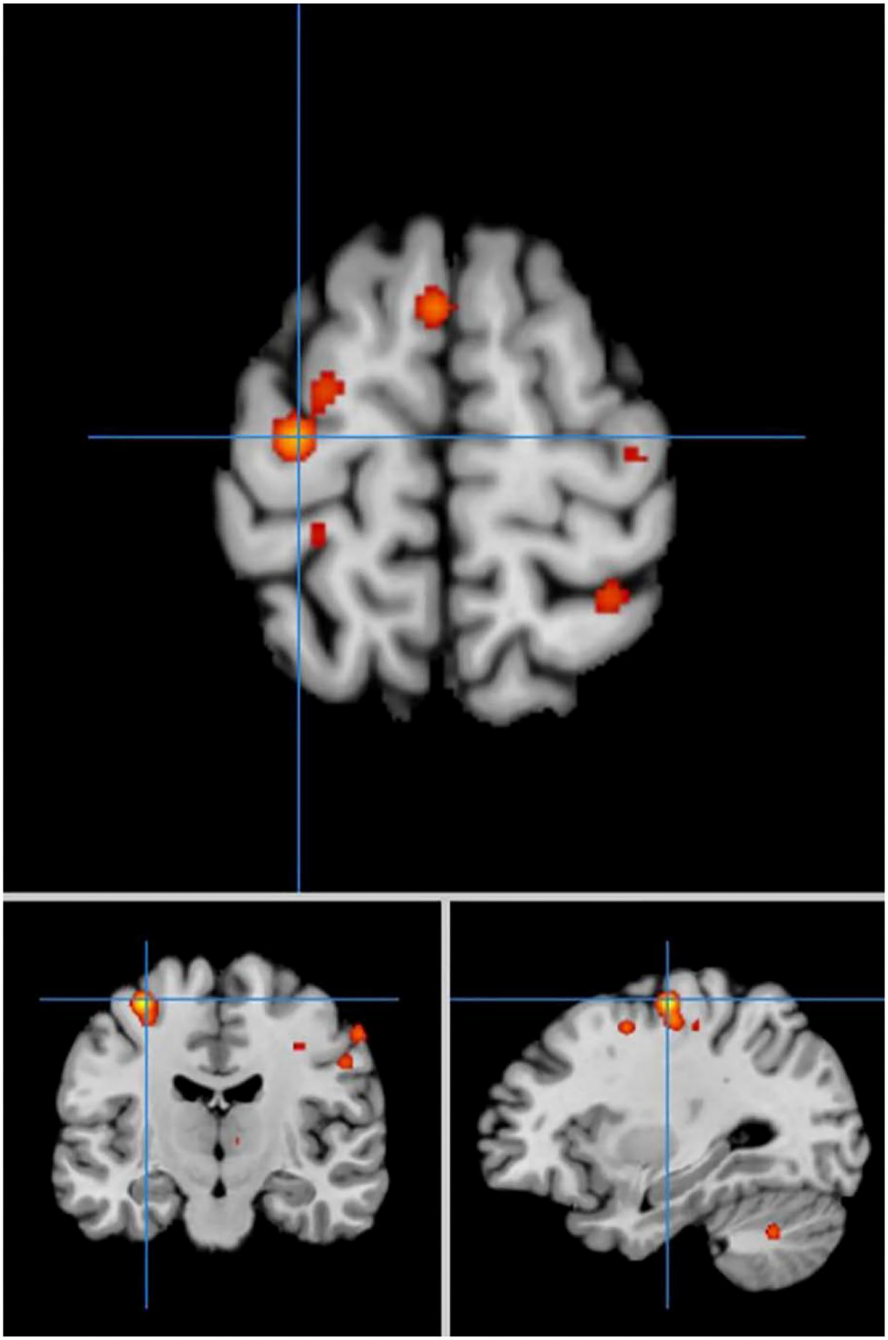
Significant clusters with PD > HC activation in the ALE meta-analysis in self-priming conditions.

In self-priming conditions, a comparison of activation in relevant brain regions during FTT performance in 135 PD patients and 112 HC participants in eight experiments revealed 55 activation points with greater activation in the PD group than in the HC group (Figure 4). We discovered 14 clusters of activation, which were located on both sides of the cingulate gyrus, inferior parietal lobule, post-central gyrus, and cerebellar folia IV; the right side of the middle frontal gyrus, inferior frontal gyrus, cerebellar folia II, and central lobule; and the left side of the superior frontal gyrus and cerebellar tonsils (Table 4).

**Table 4:** Clusters with PD > HC activation in cue-priming conditions.

**Activation cluster**	**Anatomical region**		**BA area**	**x**	**y**	**z**	**ALE**
#1	L	Superior frontal gyrus	BA 8	−24	46	40	0.006917
#2	R	Inferior frontal gyrus	BA 9	48	14	20	0.006966
#3	R	Middle frontal gyrus	BA 6	42	6	38	0.007347
#4	R	Cingulate gyrus	BA 32	18	30	28	0.008065
#5	L	Cingulate gyrus	BA 31	0	−44	36	0.007086
#6	R	Inferior parietal lobule	BA 40	40	−36	40	0.008692
#7	L	Inferior parietal lobule	BA 40	−36	−40	50	0.008392
#8	L	Cerebellar tonsils		−30	−60	−38	0.007565
#9	R	Cerebellar folia II		36	−28	10	0.007137
#10	R	Cerebellar folia IV		20	−68	44	0.008056
#11	L	Cerebellar folia IV		−24	−48	56	0.006941
#12	R	Post-central gyrus	BA 2	40	−20	58	0.007579
#13	L	Post-central gyrus	BA 5	−26	−36	66	0.00833
#14	R	Central lobule	BA 2	54	−14	34	0.007111

**Figure 5:**
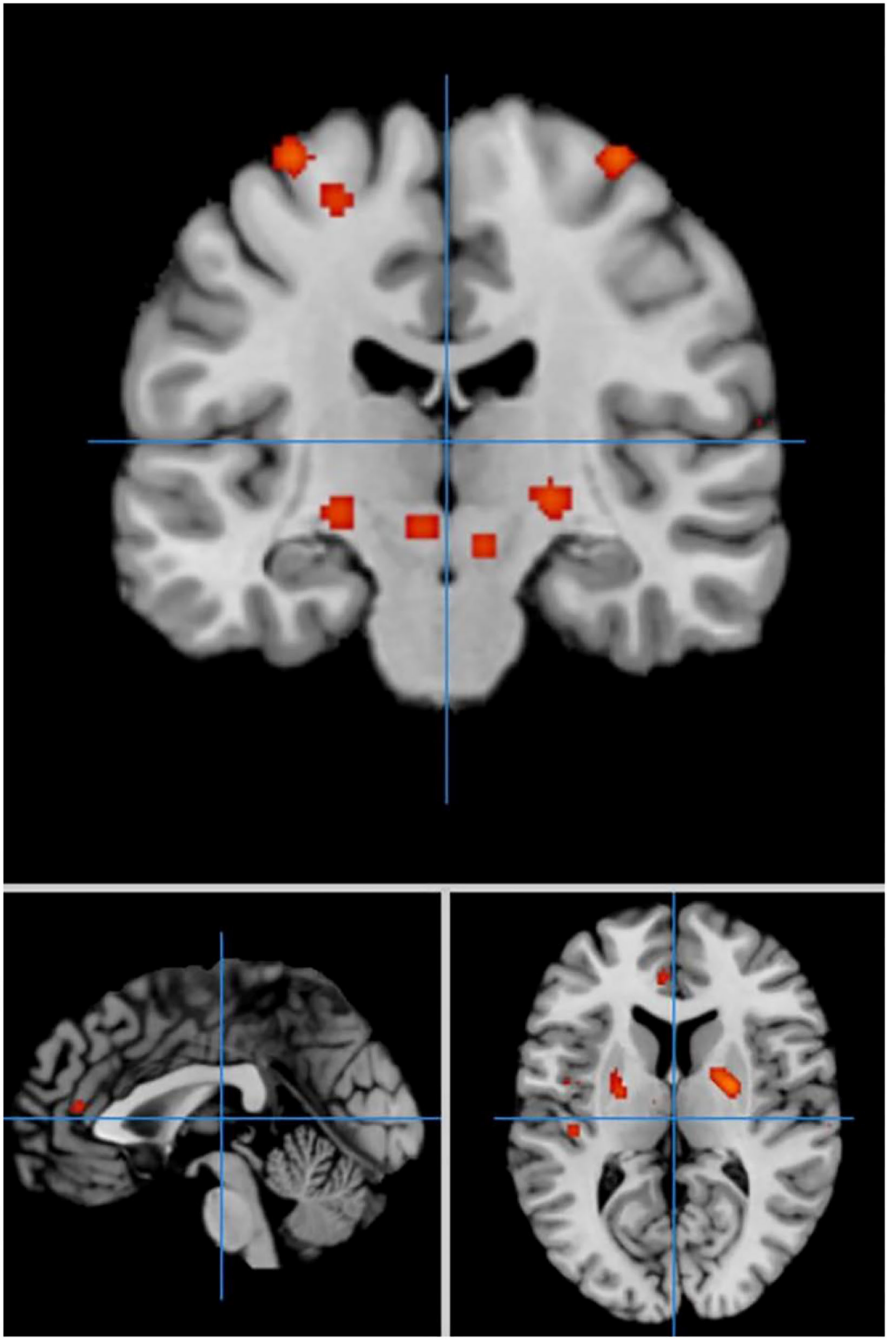
Significant clusters with HC > PD activation in the ALE meta-analysis in self-priming conditions.

Under the same conditions, 60 activation points in which HC activation was greater than PD activation were obtained (Figure 5).

We discovered 24 clusters of activation, which were located on both sides of the insula cortex, lentiform nucleus, medial frontal gyrus, superior temporal gyrus, anterior cingulate cortex, superior frontal gyrus, caudate nucleus, and precentral gyrus; the right side of the thalamus, superior parietal lobule, and precuneus; and the left side of the transverse temporal gyrus, mediodorsal thalamic nucleus, and medial geniculate nucleus (Table 5).

**Table 5:** Clusters with HC > PD activation in cue-priming conditions.

**Activation cluster**	**Anatomical region**		**BA area**	**x**	**y**	**z**	**ALE**
#1	R	Insula cortex		44	−10	−2	0.007161
#2	L	Insula cortex	13	−40	−4	12	0.007682
#3	R	Lentiform nucleus		30	0	−10	0.011021
#4	L	Lentiform nucleus		−22	−8	10	0.007789
#5	L	Transverse temporal gyrus	41	−42	−24	10	0.007331
#6	R	Red nucleus brainstem		8	−20	−14	0.00804
#7	L	Red nucleus brainstem		−6	−20	−10	0.007914
#8	R	Medial frontal gyrus	6	12	2	58	0.008337
#9	L	Medial frontal gyrus	6	−10	12	50	0.008341
#10	R	Superior temporal gyrus	42	66	−22	12	0.008037
#11	L	Superior temporal gyrus	38	−42	4	−24	0.00743
#12	R	Anterior cingulate cortex	24	12	30	−12	0.00717
#13	L	Anterior cingulate cortex	32	−6	40	12	0.010476
#14	R	Thalamus		22	−18	−4	0.007713
#15	L	Mediodorsal thalamic nucleus		−8	−12	4	0.008039
#16	L	Medial geniculate nucleus		−14	−28	−8	0.007689
#17	R	Superior parietal lobule	7	42	−57	60	0.008697
#18	R	Superior frontal gyrus	9	42	48	28	0.014485
#19	L	Superior frontal gyrus	9	−40	50	26	0.01132
#20	R	Caudate nucleus		16	0	16	0.009735
#21	L	Caudate nucleus		−14	6	18	0.008467
#22	R	Precuneus	7	10	−72	52	0.007176
#23	R	Pre-central gyrus	4	30	−14	50	0.007702
#24	L	Pre-central gyrus	4	−32	−18	68	0.008502

#### Comparisons Between the PD and HC Groups in Cue-Priming Conditions

**Figure 6:**
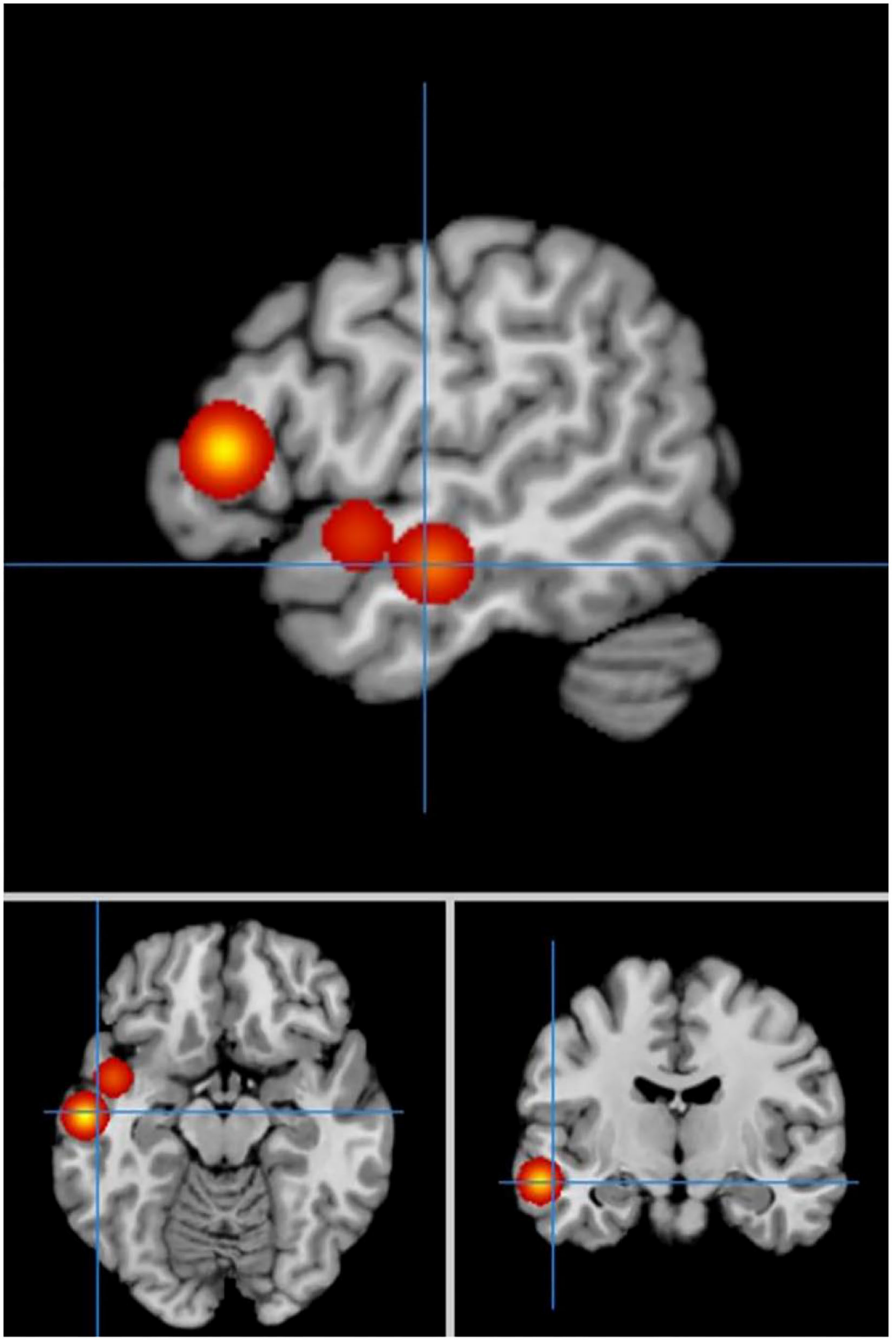
Significant clusters with PD > HC activation in the ALE meta-analysis in cue-priming conditions.

In cue-priming conditions, a comparison of activation in relevant brain regions during FTT performance in 11 PD patients and 67 HC participants revealed 51 points with greater activation in the PD than in the HC group (Figure 6). We found three clusters of activation, which were located on the left side of the superior frontal gyrus, inferior frontal gyrus, and superior temporal gyrus (Table 6).

**Table 6:** Clusters with PD > HC activation in cue-priming conditions.

**Activation cluster**	**Anatomical region**		**BA area**	**x**	**y**	**z**	**ALE**
#1	L	Superior frontal gyrus	BA 39	−20	62	20	0.006901
#2	L	Inferior frontal gyrus	BA 46	−52	30	8	0.006901
#3	L	Superior temporal gyrus	BA 38	−52	−6	−13	0.000357

**Figure 7:**
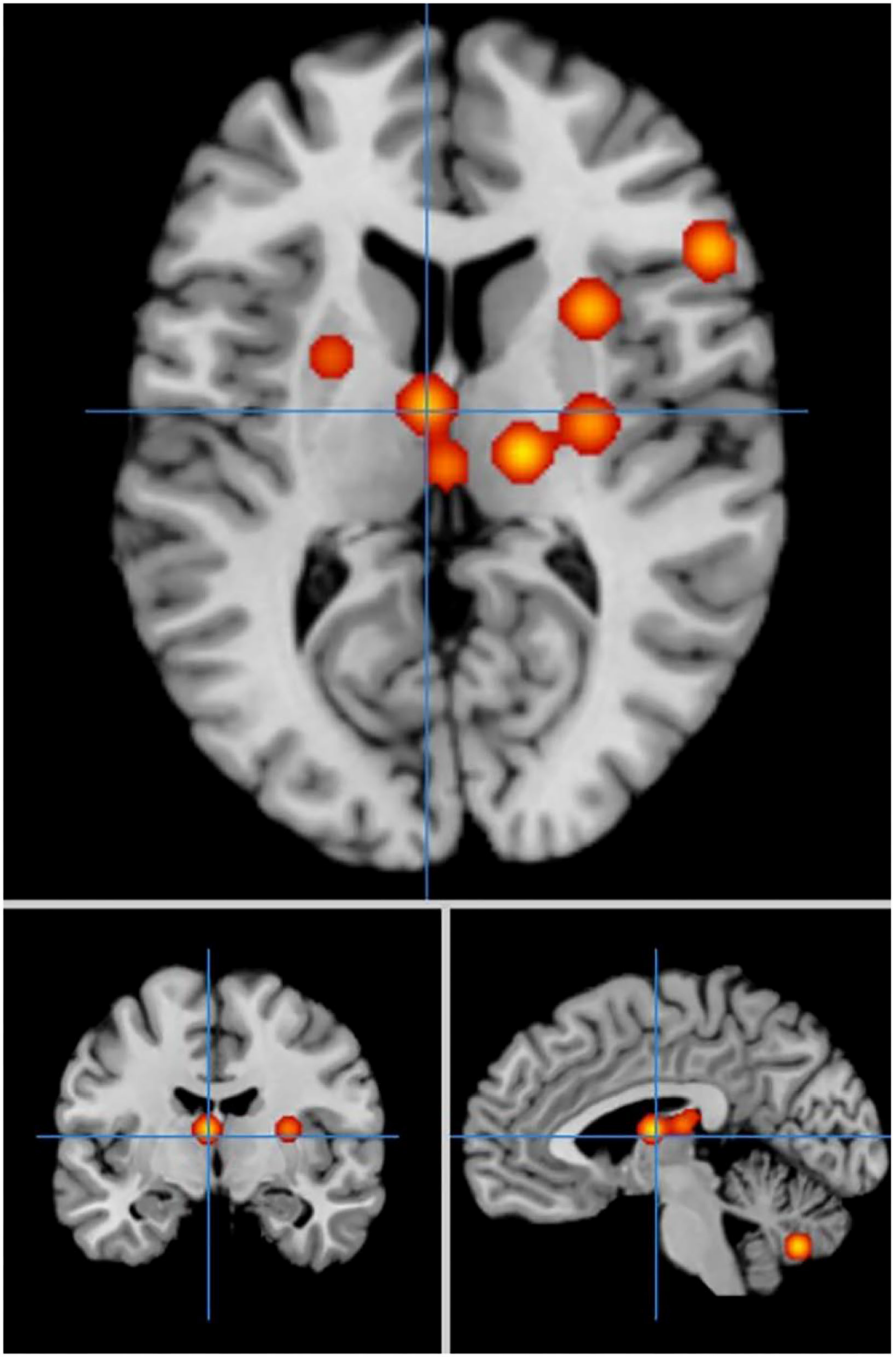
Significant clusters with HC > PD activation in the ALE meta-analysis in cue-priming conditions.

In cue-priming conditions, a comparison of activation in relevant brain regions during FTT performance in 11 PD patients and 67 HC participants revealed 15 points with greater activation in the HC group than in the PD group (Figure 7). We found 9 clusters of activation, which were located on the right side of the lentiform nucleus, claustrum nucleus, and middle frontal gyrus and on the left side of the medial frontal gyrus, thalamus, superior parietal lobule, caudate nucleus, inferior parietal lobule, and cerebellar folia II (Table 7).

**Table 7:** Clusters with HC > PD activation in cue-priming conditions.

**Activation cluster**	**Anatomical region**		**BA area**	**x**	**y**	**z**	**ALE**
#1	R	Lentiform nucleus		30	−12	10	0.006906
#2	R	Middle frontal gyrus	BA 9	46	34	24	0.006849
#3	R	Claustrum nucleus		30	12	7	0.006901
#4	L	Medial frontal gyrus	BA 6	−18	0	55	0.007787
#5	L	Thalamus		−4	−8	10	0.008051
#6	L	Superior parietal lobule	BA 7	−26	−54	50	0.007061
#7	L	Caudate nucleus		−16	8	20	0.008101
#8	L	Inferior parietal lobule	BA 40	−62	−40	28	0.008037
#9	L	Cerebellar folia II		−6	−69	−39	0.008407

## Discussion

### Neural Basis of Motor Executive Control in PD Patients During FTT Performance

The results of the present study demonstrated that during the FTT, the PD patients had a wide range of activated regions, including the middle frontal gyrus, precentral gyrus, post-central gyrus, superior parietal lobe, inferior parietal lobule, cerebellum, and basal ganglia. These results are in line with previous studies showing that when PD patients perform the FTT, brain regions associated with motor task performance, such as the primary sensorimotor cortex, supplementary motor area, basal ganglia and cerebellum, are activated (Witt et al., 2008; Liberg et al., 2013; Ruppert et al., 2020). The primary sensorimotor cortex is the key executive center not only for voluntary movements (Gerloff et al., 1998) but also for complex sequential tapping tasks (Wessel et al., 1997; Kawashima et al., 1999). The supplementary motor area has been considered essential for essential for simple autonomic movement and has also been associated with more advanced motor processing functions (Disbrow et al., 2013). Activation in the basal ganglia has been associated with simple and more complex repetitive movements (Nagano-Saito et al., 2014). In addition, regions in the cerebellum have been found to be involved in the preparation, execution, and timing of both simple and complex movements (Habas et al., 2004; Niethammer et al., 2012).

In self-priming conditions, the cingulate gyrus (BA32), inferential parietal lobule and post-central gyrus (BA2) were specifically activated in the PD patients. The cingulate gyrus has been shown to play a role in sclerometer regulation and response selection (Devinsky et al., 1995). The anterior cingulate cortex (BA24) has been associated with the somatomotor and somatosensory areas and plays a key role in attention allocation (Pardo et al., 1990; Kondo et al., 2004). In self-priming conditions, more continuous attention and movement are needed, thus activating important brain regions involved in the regulation of attention and movements, such as the cingulate gyrus and post central gyrus.

In cue-priming conditions, PD patients showed specific activations in the lingual gyrus, inferior frontal gyrus, anterior lobe, and precentral gyrus (BA4). The visual cortex is located in the lingual gyrus, which is used to detect and process visual information related to motor execution (Zeki et al., 1991; Machielsen et al., 2000; Disbrow et al., 2013); thus, it is activated to a greater extent in cue-priming conditions. The inferior frontal gyrus has been associated with finger movements (Harrington et al., 2000) and motor learning (Seitz and Roland, 1992) and participates in the inhibitory control of motor responses (Ramsey et al., 1996). The precentral gyrus is also known as the motor strip or primary motor cortex, which is mainly responsible for executing voluntary movements (Burciu and Vaillancourt, 2018). The anterior lobe of the cerebellum receives information associated with executive function from the dorsolateral pre-frontal cortex and posterior parietal lobes (Drucker et al., 2019).

In summary, FTT performance activates a wide range of brain regions involved in motor executive control. The frontal cortex of the brain showed the most activation, suggesting that it may act as the core region in finger-tapping movement. The frontal lobe of the brain is the core brain region of executive control (Zhang et al., 2017), indicating that executive control function plays a key role in FTT performance in individuals with PD.

### Brain Activation Contrasts Between PD Patients and HCs in Self-Priming Conditions

In self-priming conditions, the PD patients showed more activation in the frontal lobe areas, inferior parietal lobule and cerebellum than the HC participants. In the HC groups, the activation in the superior parietal lobule, insula cortex and basal ganglia was more significant.

A great deal of evidence suggests that the coordination of goal-driven behavior is supported by the frontoparietal network (FPN) (He et al., 2007; Marek and Dosenbach, 2018). The dorsolateral pre-frontal cortex (BA 9) and ventrolateral pre-frontal cortex (BA 45) are involved in regulating movement and behavior through inhibitory control. The parietal cortex plays a role in selecting and monitoring motion sequences (Deiber et al., 1991). It is involved in time perception of motion sequences to ensure that each movement occurs after the successful completion of the previous movement (Sirigu et al., 1996; Macar et al., 2002; Bueti and Walsh, 2009). The cerebral cortex, which includes areas such as the pre-frontal and parietal cortices, the dorsal cingulate cortex and the insula, may constitute a reactive inhibition pathway, playing a role in inhibitory control (Macar et al., 2002; Meyer and Bucci, 2016).

In self-priming conditions, the PD patients showed activation in the cerebellum, including the central lobule, cerebellar folia II, cerebellar folia VI and cerebellar tonsils. The mechanisms underlying the wide range of activation in cerebellar areas remain unclear. It is possible that activation of some specific cerebellar areas compensates for impaired basal ganglia function in PD patients (Kübel et al., 2018). The activity of cerebellum was increased during automated movements, which may reflect compensatory function of cerebellum. The activation of the supplementary motor area (including the promoter cortex and the association area) in PD patients is relatively insufficient in self-priming conditions. It is possible that this region is a key brain region for initiating movement, especially for internally generated voluntary movement (Tanji and Hoshi, 2001; Disbrow et al., 2013). Insufficient activation of the supplementary motor area may also contribute to deficits in timing and generating movements (Simmonds et al., 2008; Jacobs et al., 2009).

In self-priming conditions, the regions that were more activated in HCs reflect the pathological structural regions in PD patients. These areas are also important targets for the development of interventions for PD treatment “or” for the development of interventions or PD treatment (e.g., exercise-based interventions). The lentiform nucleus and the caudate nucleus within the basal ganglia are widely acknowledged as key sites of PD pathology (Albani et al., 2001). The thalamus acts as the central relay station of the brain, filtering and transmitting sensory input to the cerebral cortex, and is a key node in the cerebellum-thalamus-prefrontal cortex pathway (Jech et al., 2012). The parietal lobe is an important sensory area that is associated with executive function. Insufficient activation or reduced connectivity in these areas may result in decreased motor executive control function and even motor impairment in PD patients (Tinaz et al., 2016). When PD patients perform self-priming exercises, not only the frontal lobe and parietal lobe show abnormal activities but also the interactions within the motor network are disrupted. The reduction in psychomotor functional connectivity (especially in cortico-basal ganglia and basal ganglia-cerebellum loops) may lead to impairments in automatic movements in PD patients (Palomar et al., 2013; Smittenaar et al., 2013). PD patients may need to increase connectivity within the cortico-cerebellum loop to compensate for the dysfunction of the basal ganglia to properly perform the task in the self-priming condition.

### Brain Activation Contrasts Between PD Patients and HCs in Cue-Priming Conditions

In cue-priming conditions, the PD patients showed more activation on the left side in the superior frontal gyrus, inferior frontal gyrus, and superior temporal gyrus. In the HCs, activation in the cerebellum, thalamus, caudate nucleus, superior parietal lobule, inferior parietal lobule, right middle frontal gyrus, insula cortex and basal ganglia was more significant.

When the FTT was performed with external cues, the PD patients needed to coordinate finger movements with the external stimulus signals. In cue-priming conditions, the superior frontal gyrus and superior temporal gyrus in PD patients showed greater activation. These activations may reflect compensatory mechanisms. Past studies have shown that the cortical-striato-thalamic-cortical circuit is a key loop for motor initiation and inhibition and maintains the balance between activation and inhibition in the motor circuit (Hacker et al., 2012; Peters et al., 2016). External cues may enhance the input of the frontal region in the executive and inhibitory functions related to motor tasks in PD patients, thus compensating for the functional deficits of the basal ganglia in the selection and execution of motor tasks and improving the imbalance of activation and inhibition circuits.

The parietal lobe plays an important role in sensory-motor transformation and visual guidance of movement (Wu and Hallett, 2005). The posterior parietal cortex is associated with motoring highly integrated tasks, including task switching, visually guided motor planning, and attentional control (Macaluso et al., 2000; Liston et al., 2006). Insufficient activation in the parietal lobes of PD patients leads to impaired functions in motor planning. However, external cues may mobilize the activation of the visual area of the superior temporal gyrus, which is also involved in motor executive control, and thus, improve motor performance in PD patients (Vikene et al., 2019). This result indicates that in addition to the parietal lobe. Other brain regions within sensory pathways may have similar functions.

The activation levels in the cerebellar regions, especially in the cerebellar folia II, were lower in the PD group than in the HC group under cue-priming conditions. This result is consistent with previous studies on bimanual tasks (Islam et al., 2011) and the viscometric predictive tracking task in PD patients (Turner et al., 2003). Interactively in the cerebellar areas may imply that PD patients have difficulty achieving sensorimotor integration of finger movements with external stimulus signals (Jueptner and Weiller, 1998; van Donkelaar et al., 2000; Lewis et al., 2013). However, some studies have reported over-activation in the cerebellum in PD patients during cue-priming FTT and thumb pressing (Mak et al., 2016). Due to the limited numbers of studies examining cue-priming conditions included in the current meta-analysis, how external cues impact the function of cerebellar regions needs further investigation. Increasing evidence has shown that the role of the cerebellum in PD is complicated (Strick et al., 2005; Bostan et al., 2010; Stoodley et al., 2012). Different areas of the cerebellum may show different responses to stimulation and action execution.

## Strength and Limitations

The present study provides a quantitative summary of past imaging studies with the FTT in PD patients, with a focus on the impact of the priming condition on the distribution of activation across brain regions. The study demonstrates that the two priming conditions yield some divergence in areas activated in PD patients. The cue-priming manipulation alters the brain regions that are activated and its functional expression during motor execution. It is worth discussing the potential influence of external cues on motor executive control in PD patients. In cue-priming conditions, brain activity in regions associated with perceptual processing and inhibitory control was enhanced. The results of the current study provide a neuropathological basis for using external cues in motor executive training in treating mild to moderate PD.

The present study has several limitations. First, although coordinate-based meta-analyses (CBMA), such as ALE, can assess whether the convergence between reported coordinates in the brain is statistically higher (Eickhoff et al., 2012), they cannot explain whether the activated coordinates are a direct result of the task itself or whether the brain regions are functionally coupled with other regions. To investigate the effective connectivity of brain regions, future studies can adopt methods such as the dynamic causal model (DCM) (Stephan et al., 2007). Second, only 15 studies (with 160 foci), which had small sample sizes, fulfilled the inclusion criteria and were included in the ALE meta-analysis. This limitation may have reduced the power of the analysis (Eickhoff et al., 2016). Future work with larger samples is needed to develop better interpretations of activated regions during FTT performance in PD patients. In addition, due to a lack of research evaluating cue-priming conditions with the FTT in clinical studies, this meta-analysis simply distinguished the experimental conditions into self-priming and cue-priming. With an increase in the number of relevant studies, future meta-analyses can refine the attributes of priming conditions (e.g., visual, auditory, tactile) or a combination of these sensory cues to examine the brain activation of motor executive control in PD patients.

## Conclusion

In summary, using ALE meta-analysis, this study found that there was significant activation in the middle frontal gyrus, precentral gyrus, post-central gyrus, superior parietal lobe, inferior parietal lobule, cerebellum, and basal ganglia during FTT performance in PD patients. In self-priming conditions, PD patients had less activation in the parietal lobe and insular cortex than the HCs, however, cerebellar areas were overactivated. In cue-priming conditions, the cerebellum and frontal-parietal areas were less activated, and the superior frontal gyrus and superior temporal gyrus were overactivated in PD patients. Our study illustrates that the cue-priming manipulation affects the distribution of activity in brain regions involved in motor control and motor performance in PD patients. In cue-priming conditions, brain activity in regions associated with perceptual processing and inhibitory control was enhanced. The sensory motor areas associated with attention and motor control were impaired. In the design of future interventions for PD and other clinical movement disorders, cue-priming manipulations can be utilized in designing interventions (e.g., exercise-based interventions) for PD patients.

## Data Availability Statement

The original contributions presented in the study are included in the article/supplementary material, further inquiries can be directed to the corresponding author.

## Author Contributions

XX contributed to the supervision of the meta-analysis and provided the methodology expertise. JL conceptualized the project, organized the study selection, data extraction, performed the data analysis, and visualized the results. ZL and ZD contributed to the critical review of relevant literature. NZ and XQ contributed to the selection and coding procedures. JL, ZL, and ZD contributed to the discussion of the results and drafting of the final manuscript. All authors critically revised the manuscript and approved the final version.

## Funding

This research was supported by National Natural Science Foundation of China (Grant no. 81971661), Hubei Superior Discipline Group of Exercise and Brain Science from Hubei Provincial Department of Education.

## Conflict of Interest

The authors declare that the research was conducted in the absence of any commercial or financial relationships that could be construed as a potential conflict of interest.

## Publisher's Note

All claims expressed in this article are solely those of the authors and do not necessarily represent those of their affiliated organizations, or those of the publisher, the editors and the reviewers. Any product that may be evaluated in this article, or claim that may be made by its manufacturer, is not guaranteed or endorsed by the publisher.

## Footnotes

^1^The Unified Parkinson's Disease Rating Scale (UPDRS) was introduced in 1987, which is used to monitor the course of Parkinson's disease and the degree of disability (Helme, 1987).

^2^The Hoehn and Yahr scale (H&Y scale) was originally developed in 1967 and is used to measure the level of disability in PD (Hoehn and Yahr, 1967).
